# Multigenerational daytime-restricted feeding exacerbates irradiation-induced hepatic DNA damage and aging in mice

**DOI:** 10.3389/fnut.2026.1917104

**Published:** 2026-09-09

**Authors:** Lanqing Sun, Kai Huang, Huali Cheng, Yike Wang, Xi Yang, Wenjun Zhang, Jun Qian, Qiaocheng Zhai

**Affiliations:** 1 Department of Laboratory Medicine, Affiliated Hospital of Jiangnan University, Wuxi, Jiangsu, China; 2 School of Food Science and Technology, Jiangnan University, Wuxi, Jiangsu, China; 3 Orthopaedic Institute, Wuxi Ninth People’s Hospital Affiliated to Soochow University, Wuxi, Jiangsu, China; 4 Jiangsu Key Laboratory of Neuropsychiatric Diseases and Cambridge-Su Genomic Resource Center, Medical School of Soochow University, Suzhou, Jiangsu, China; 5 Department of Orthopaedics, The Second Affiliated Hospital of Soochow University, Suzhou, Jiangsu, China; 6 Department of Orthopaedics, Quzhou People’s Hospital, The Quzhou Affiliated Hospital, Wenzhou Medical University, Quzhou, Zhejiang, China

**Keywords:** cellular senescence, circadian, DNA damage, multigeneration, time-restricted feeding

## Abstract

**Introduction:**

While feeding timing profoundly impacts hepatic circadian rhythms due to the liver’s high sensitivity to dietary signals, its cumulative, intergenerational consequences on vulnerability to environmental stress and stress-induced senescence remain largely unknown. Therefore, this study aimed to investigate the intergenerational effects of multigenerational daytime-restricted feeding (DRF) on the hepatic DNA damage response and liver health in a mouse model.

**Methods:**

A multigenerational DRF mouse model was established, where mice from the F0 to F10 generations were subjected to a feeding window restricted to 6 hours during the light phase (Zeitgeber time, ZT2ZT8). Hepatic transcriptional rhythms were analyzed, and mice were exposed to 1 Gy X-ray irradiation to induce DNA damage. DNA damage was subsequently assessed via γH2AX immunofluorescence, and cellular senescence was evaluated by senescence-associated beta-galactosidase (SA-β-gal) staining alongside p21 expression.

**Results:**

Multigenerational DRF disrupted the circadian transcriptional rhythms of key DNA repair genes (e.g., *Wrnip1, Nabp2, Fan1*), with a trend towards downregulation in F10 livers. Following irradiation, F10 mice exhibited significantly increased γH2AX foci, indicating exacerbated DNA damage. Furthermore, these mice demonstrated elevated p21 expression and a higher number of SA-β-gal-positive senescent cells compared to earlier generations, whereas apoptosis markers (BAX, BCL2) remained unchanged.

**Conclusion:**

Persistent multigenerational DRF impairs the circadian regulation of DNA repair genes and sensitizes the liver to genotoxic stress, leading to increased DNA damage and accelerated cellular senescence. These findings underscore the significant intergenerational impact of dietary timing on the hepatic DNA damage response and highlight the critical importance of meal timing in maintaining liver health and preventing age-related pathologies.

## Introduction

Circadian clock is a hierarchical system including central clock located in the suprachiasmatic nucleus (SCN) and clock in peripheral organs (1). About 43% of all protein coding genes displayed transcriptional rhythm in mice (2). Irregular eating patterns such as skipping breakfast and night-time eating are detrimental to individual health (3–7). Similar to the well-documented intergenerational impacts of obesogenic diets, persistent mistimed feeding has been shown to exert profound physiological effects on murine offspring (8–11).

Time-restricted feeding (TRF) refers to a dietary pattern in which feeding is restricted to a specific time window, with fasting during the remaining period (12). Daytime-restricted feeding (DRF) exerts robust effects on hepatic gene expression rhythms independently of the suprachiasmatic nucleus and hepatic core clock genes, and liver is more sensitive to dietary signals than other peripheral organs (13–16). Utilizing a multigenerational DRF mouse model, our prior work established the critical role of dietary timing in modulating the hepatic endoplasmic reticulum stress response across generations (11). However, further exploration is needed to investigate more intergenerational effects of dietary timing changes on hepatic stress response function.

DNA damage response (DDR) involving both cell cycle checkpoints and DNA repair are under circadian control. CLOCK: BMAL1 complex has been reported to modulate the expression of essential genes controlling cell cycle progression and responses to genotoxic stress, which drives rhythmic expression of DDR genes (17, 18). Apart from regulating gene expression, core clock protein such as PER1 forms a complex with DDR factors in response to genotoxic stress. Previous report has revealed that PER1 interacted with DNA double-strand-breaks (DSBs) response kinase ATM and checkpoint kinase CHK2 (19). Moreover, PER1 has been found to mediate intergenerational effects on DNA repair. Exposure of parental marine medaka to benzopyrene inhibited PER1 expression in offspring through DNA methylation, leading to impaired DNA repair function and severe DNA damage in the offspring (20). Interestingly, DRF could modulate diurnal expression of DNA repair genes and alter the sensitivity to UVB-induced DNA damage (21). These observations confirm the role of the circadian clock in regulating DNA repair. Aging is accompanied by a continuous onslaught of DNA damage stemming from endogenous metabolic activities, cell division, and exogenous environmental stressors (22). Compounding this issue, the efficacy of DNA repair machineries deteriorates over time. As a result, the progressive accumulation of genomic lesions has been firmly established as a defining hallmark of cellular senescence and aging (22). In the context of liver pathology, ageing is widely recognized as a critical contributor to the onset of acute and chronic hepatic diseases (23). Given the strong influence of DRF on hepatic clock, we hypothesized that persistent alterations in dietary timing may have intergenerational effects on the susceptibility of the liver to DNA damage.

Here, we constructed multigenerational DRF mouse model and found that a cluster of hepatic genes related to DNA repair exhibited a trend of transcriptional rhythm loss from F2 to F10 mice. Moreover, multigenerational DRF exacerbated the X-ray irradiation-induced DNA damage and cellular senescence in the liver of F10 mice. Our study provides new insights into the intergenerational effects of DRF on hepatic DNA damage response and underscores the importance of diet timing in liver health.

## Materials and methods

### Animals

Multigenerational DRF model was constructed as previously described (11). All mice were housed in the same specific pathogen-free (SPF) facility under strictly controlled conditions: a constant 12:12 light–dark cycle (lights on at 8:00 a.m.), temperature (22 ± 1 °C), humidity (50 ± 10%). Mice were group-housed (2–5 animals per cage) in standard ventilated cages with corn cob bedding, which was changed weekly. To accurately quantify food consumption, mice were individually housed in single cages. The daily food intake was recorded per mouse, and no significant differences were observed between the DRF and ad libitum groups (11). 8 weeks old wild-type (WT) male and female mice were mated to generate F0 offspring and F0 were assigned to daytime restricted feeding (DRF) from weaning. Food was available to DRF mice for a 6-h period starting at ZT0 (ZT = Zeitgeber time, ZT0 refers to lights on, ZT12 refers to lights off). Then, F0 male and female mice were selected randomly to mate and produce F1 offspring. The F2 to F10 generations were similarly created. Since F0 generation, all mice were maintained on DRF throughout their lifespan prior to experimental euthanasia. This multigenerational study was designed to compare four key groups: F2, F5, and F10 generations of mice subjected to lifelong daytime-restricted feeding, with the F2 group serving as the internal control. Age-matched wild-type mice fed *ad libitum* were included as an external standard control. The experimental unit for all analyses was the individual animal. All experiments in this study were conducted using male mice to avoid potential variability associated with the female estrous cycle. Mice were randomly selected from the entire cohort of that generation using a random number generator. This random selection ensured that each mouse had an equal probability of being chosen, minimizing selection bias. To minimize potential confounders, cage positions were rotated regularly and key procedures (e.g., irradiation) were performed in an interleaved manner across groups. The DNA damage model was adapted from previous studies (24). Mice were received whole-body irradiation using an X-ray biological irradiator (RS-2000 Pro, Rad Source Technologies) at Zeitgeber time 8 (ZT8, immediately at the end of the 6-h feeding window). A total dose of 1 Gy was administered at a dose rate of 1 Gy/min. 30 min after radiation, mice were euthanized and liver samples were collected. The 30-min post-irradiation time point was selected based on established literature showing that the DNA damage repair response initiates within minutes and is actively ongoing by 30 min (25–27) (e.g., γH2AX foci formation and early repair protein recruitment). Furthermore, to evaluate the DNA repair efficiency and kinetics, a 6-h post-irradiation time point was also included to track the resolution of genomic lesions. This time point therefore captures the acute phase of damage recognition and the initial capacity for repair activation, rather than later resolution. Our study specifically aimed to assess whether multigenerational DRF affects this initial hepatic responsiveness to genotoxic stress. For senescence-associated beta-galactosidase (SA-β-gal) staining, liver samples were collected 1 month after irradiation. In total, approximately 60 male C57BL/6 mice were used across all experiments described in this study. Mice entered the experiments at 8–10 weeks of age, with a body weight of approximately 25 grams at the start. The sample size per group (*n* = 3–4) was selected to robustly assess the primary intergenerational phenotype across multiple generations while balancing experimental scale. Our sample size is justified by: (1) a large, pilot-validated effect size (*f*^2^ = 0.42) that exceeds the threshold for reliable detection in animal studies; (2) > 80% statistical power to detect predefined differences; (3) zero attrition; and (4) compliance with ethical principles of minimizing animal usage. All calculations align with the methods and recommendations in the referenced article (28). At the designated endpoints, mice were euthanized using a graded CO₂ protocol with medical-grade CO₂ (purity ≥99.5%). The chamber concentration was first raised to 30%, then rapidly increased to 70% within 5–10 s, and maintained for at least 5 min after breathing ceased to ensure death. Death was verified by the absence of a heartbeat and cessation of breathing, followed by secondary confirmation via physical stimuli (toe pinch) to ensure no reflex response before tissue collection. All efforts were made to minimize the number of animals used and to alleviate potential suffering. The animal study was reviewed and approved by the Animal Care and Use Committee of the Cambridge-Su Genomic Resource Center, Soochow University (Approval Numbers: YX-2021-2, approval date: December 25, 2020). The reporting of this animal study conforms to the ARRIVE 2.0 guidelines (29). Animal care and all experimental procedures were conducted in accordance with the Guide for the Care and Use of Laboratory Animals (30).

### Inclusion/exclusion criteria and data analysis

No formal, pre-defined (*a priori*) criteria were established for excluding animals or specific data points. All offspring weaned into the multigenerational DRF protocol were included in the study cohort. The only basis for removing an animal was the onset of a pre-defined humane endpoint (e.g., severe weight loss >20%, moribund state), as required by our animal ethics protocol. In this study, no animals, experimental units, or data points were excluded from the final analysis. All collected data from the included animals are presented.

### Quantitative RT-PCR

Total RNA isolated from liver tissue was reverse transcribed, followed by quantitative PCR with SYBR Green detection. Primer sequences used are as follows:

**Table 1:** 

Genes	Forward primers 5′–3′	Reversed primers 5′–3′
*Actin*	CTGTCCCTGTATGCCTCTG	ATGTCACGCACGATTTCC
*Wrnip1*	GCGAGATGTCATAAAGCAGGCTC	CAGTAGTCGCACCAATCAGCGT
*Fan1*	GAGTGGCTTTGACCAAGGGA	CAAATCCAGTGGGGATGCCT
*Nabp2*	GTAAAGTGGCAGACAAAACGGGC	TACAGTGTCAGACAACCTTTGAAC
*Ino80*	GCCAGCAGAAATGGCAAACCTC	CAGATAGCTCTGATGGCTTGTCC
*Prpf19*	GATTCTCGCTCTGGACCTGTGT	ACCACACTGGTGACCTTCTTGG
*p21*	GAGGATGTGGGCACGGTGGT	CCCTGATCGTTGGCTATGAT
*Bax*	AGGATGCGTCCACCAAGAAGCT	TCCGTGTCCACGTCAGCAATCA
*Bcl2*	CCTGTGGATGACTGAGTACCTG	AGCCAGGAGAAATCAAACAGAGG
*Il6*	TACCACTTCACAAGTCGGAGGC	CTGCAAGTGCATCATCGTTGTTC
*Tnfa*	GGTGCCTATGTCTCAGCCTCTT	GCCATAGAACTGATGAGAGGGAG

### Western blot

Liver protein was extracted using RIPA buffer with protease inhibitors, followed by SDS-PAGE electrophoresis and transferred to PVDF membranes. Membranes were probed with primary antibodies after blocking and maintained at 4 °C overnight. Antibodies used include P21 (10355-1-AP; Proteintech; RRID: AB_2077682), BAX (2772; Cell Signaling; RRID: AB_10695870), BCL2 (3498; Cell Signaling; RRID: AB_1903907), CHK1 (2360; Cell Signaling; RRID: AB_2080320), p-CHK1 (2,348; Cell Signaling; RRID: AB_331212), ACTIN (A5441; Sigma Aldrich; RRID: AB_476744). After incubated with horseradish peroxidase (HRP)-conjugated secondary antibodies for 1 h at room temperature, membranes were visualized with an ECL reagent. Band intensity was quantified using ImageJ software. The integrated density of each target protein band (e.g., p21) was normalized to the integrated density of the corresponding ACTIN band.

### Immunofluorescence assay and SA-β-gal staining

Liver tissues were immediately snap-frozen in liquid nitrogen and embedded in OCT compound. Cryosections (6 μm) were prepared. Following fixation with 4% PFA (20 min), sections were blocked with 5% BSA (1 h, RT). Then, liver sections were incubated with anti-γH2AX antibody (9,718; Cell Signaling; RRID: AB_2118009) in 5% BSA for 2 h at room temperature. After incubating with anti-rabbit Cy3 secondary antibody (ab97075; abcam), sections were analyzed by microscope. Images were acquired using a confocal microscope. Foci counting was performed using ImageJ software. Individual, bright punctate signals distinctly above the diffuse nuclear background were counted as a single focus. For each mouse, foci were counted in approximately 100 randomly selected hepatocytes across multiple non-overlapping fields. The average number of foci per cell was calculated for each animal.

For SA-β-gal staining, after fixed in 4% paraformaldehyde and 0.2% glutaraldehyde for 5 min, sections were immersed overnight in SA-β-gal staining solution according to the previously reported protocol (31). Sections were counterstained with nuclear fast red and visualized under microscope. The number of SA-β-gal positive cells (displaying perinuclear blue staining) and the total number of cells were counted manually in each field using ImageJ. Cells exhibiting a clear, granular blue cytoplasmic precipitate were scored as positive. For each mouse, the percentage of positive cells was determined from counts across approximately 600 hepatocytes in multiple, randomly selected fields.

### Rhythmicity and functional enrichment analysis

The raw sequencing data supporting the rhythmicity analysis in this study are available in the NCBI SRA repository under BioProject PRJNA932189, and the data from BioSample accessions SAMN33179963, SAMN33179964, and SAMN33179965 under Project PRJNA932189 were used (11). The re-analyzed RNA-seq data were generated from liver samples collected every 4 h across a 24-h cycle (6 time points: ZT0, ZT4, ZT8, ZT12, ZT16, ZT20). For each generation (F2, F5, F10), *n* = 2 biological replicates (mice) were used per time point. Transcriptional rhythms were identified using the meta2d algorithm from the MetaCycle R package (v1.2.0), which is specifically designed for omics time-series data (32). A gene was considered to exhibit a significant circadian rhythm if its adjusted *p*-value (meta2d output) was <0.05. No amplitude cutoff was applied beyond this statistical threshold. In this study, we define ‘loss of rhythmicity’ as a change in statistical significance: a gene that is rhythmically expressed (*p* < 0.05) in an earlier generation (e.g., F2) becomes non-rhythmic (*p* > 0.05) in a later generation (e.g., F10). Gene Ontology (GO) enrichment analysis for genes losing rhythmicity was performed using Metascape (metascape.org). The analysis was run with default parameters. Significantly enriched terms were selected based on Log10(*p*) < −3 (equivalent to *p* < 0.001), a minimum count of 5, and an enrichment factor >1.5.

### Quantification and statistical analysis

**Figure 1:**
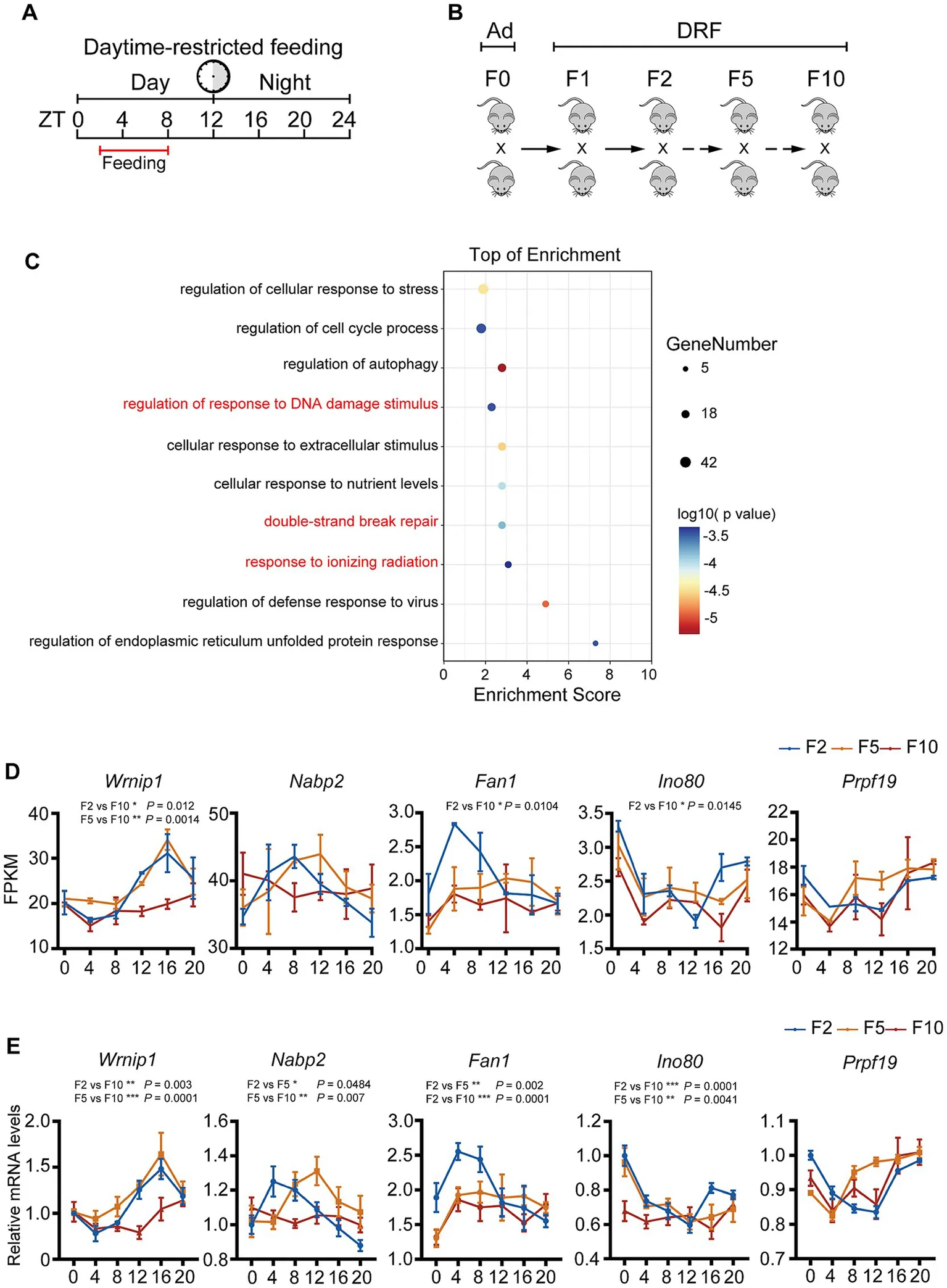
Multigenerational DRF disrupts transcriptional rhythm of DNA repair-associated genes. **(A)** The DRF schematic, with the mouse feeding time restricted to ZT2–ZT8. **(B)** Schematic of multigenerational DRF model. **(C)** Pathway enrichment analysis of rhythm-loss genes from F2 to F10. **(D)** Expression patterns of representative rhythm-loss genes involved in DNA damage response [for each generation (F2, F5, F10), *n* = 2 biological replicates (mice) were used per time point]. **(E)** Diurnal mRNA expression profiles of representative DNA repair genes validated by independent time-course qPCR (*n* = 2 biological replicates per time point). Please note that the loss of rhythmicity depicted in panels **(D,E)** constitutes a preliminary observation due to the limited biological sample size utilized for rhythmicity analysis. All error bars represent SD.

To minimize bias, a stringent blinding procedure was implemented. After tissue collection, all samples were de-identified with random codes by an independent researcher. All subsequent quantitative analyses including γH2AX foci counting, SA-β-gal-positive cell quantification, and Western blot band densitometry were performed by experimenters blinded to group assignments (generation and treatment). The group identities were only revealed after data analysis was complete. Data represent mean values ± standard deviation. The analysis of circadian transcriptional rhythms over time and across generations in Figure 1D was performed using a two-way ANOVA. All other group comparisons were performed using a one-way ANOVA. The normality of data distribution for each group was assessed using the Shapiro–Wilk test. The homogeneity of variances was tested using the Brown–Forsythe test, which is robust to departures from normality. Following a significant ANOVA result (*p* < 0.05), Tukey’s Honestly Significant Difference (HSD) *post-hoc* test was applied for all pairwise comparisons. This test inherently corrects for multiple comparisons. Significance levels throughout the figures and text have been standardized as follows: *p < 0.05, **p < 0.01, ***p < 0.001. Non-significant (ns) comparisons are explicitly marked where relevant. GraphPad Prism 9 software was employed for all statistical evaluations.

## Results

### Multigenerational DRF disrupts transcriptional rhythm of DNA repair-associated genes

To construct multigenerational DRF model, the feeding period of mice is restricted to ZT2-ZT8 (ZT = Zeitgeber time, ZT0 represents light onset, ZT12 represents light off), and mice were under DRF all life from weaning (Figures 1A,B). Our previous work indicated that multigenerational DRF had minimal intergenerational (across successive generations that are all exposed to the intervention) effects on mice physiology including body weight gain and energy expenditure (11). However, at the molecular level, intergenerational differences in hepatic transcriptional rhythm of the liver were observed in mice. From F2 to F10, hundreds of genes lost transcriptional rhythm without significant changes in the expression of core clock genes (11). GO enrichment analysis showed that genes losing rhythmic expression were mainly related to DNA damage response (Figure 1C). Typically, *Wrnip1*, *Nabp2*, *Fan1*, *Ino80*, and *Prpf19*, which exert DNA repair function (21, 33–36), lost transcriptional rhythm and exhibited a trend of downregulated expression in the liver of F10 mice (Figure 1D). To methodologically validate the sequencing data, targeted time-course qPCR analysis was performed on the identical RNA samples. Although this cross-platform assessment confirmed the technical repeatability of the blunted diurnal expression patterns for these DNA repair genes in the F10 generation (Figure 1E), it does not enhance biological statistical power. Given that the rhythmicity analysis utilizes a curve-fitting algorithm (MetaCycle) on a restricted biological sample size (*n* = 2 per time point), the statistical power remains limited. Consequently, the observed loss of rhythmicity for these genes should be interpreted as a preliminary finding. These results suggest hepatic DNA damage response function might be impaired by multigenerational DRF.

### Multigenerational DRF exacerbates irradiation-induced DNA damage

**Figure 2:**
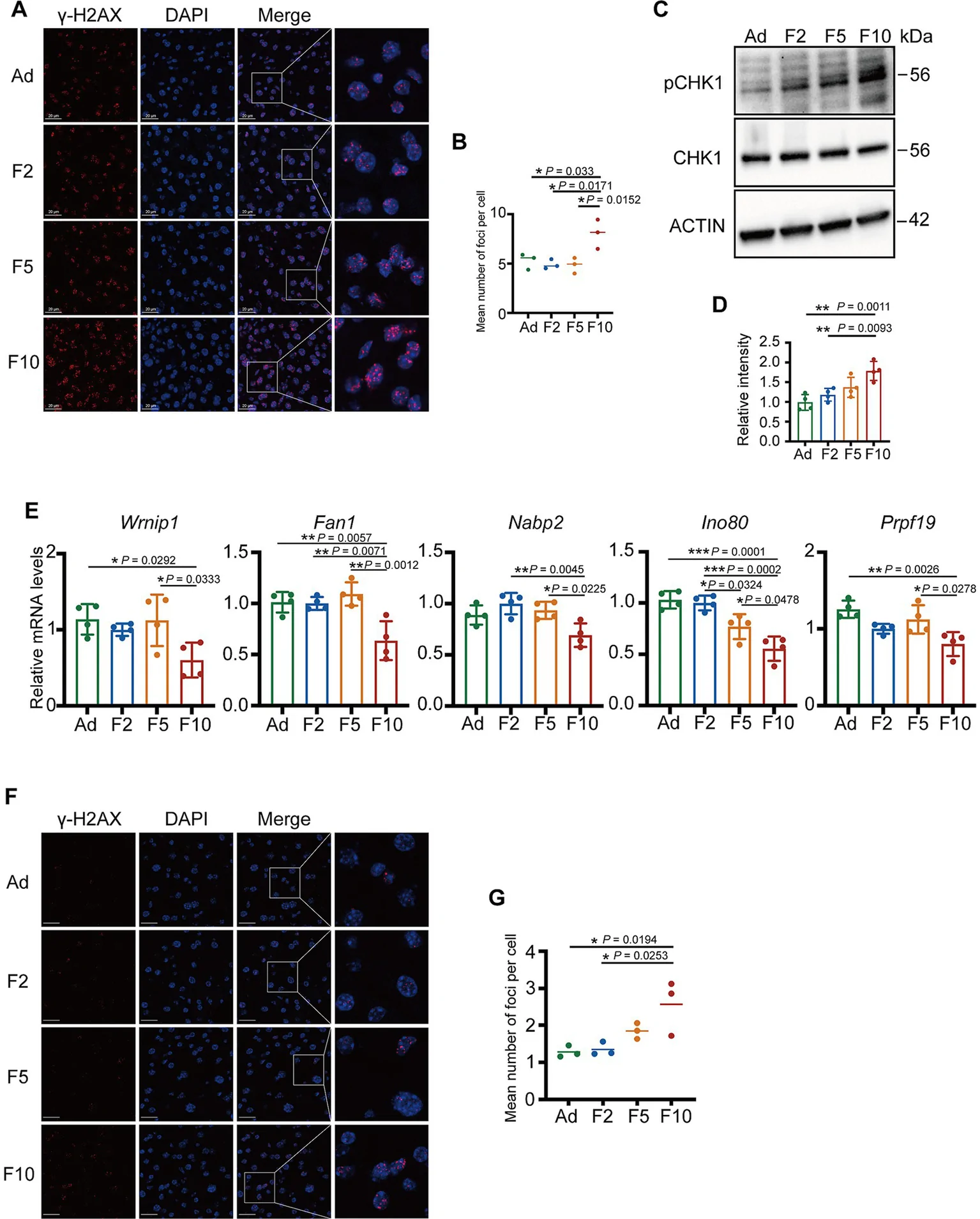
Multigenerational DRF exacerbates irradiation-induced DNA damage. Liver tissues were sampled 30 min after X irradiation. **(A)** Representative γH2AX staining (red) of mouse liver (*n* = 3). Nuclei were stained with DAPI (blue); scale bar, 20 μm. **(B)** Quantification of γH2AX foci per nucleus (*n* = 3). **(C)** Representative Western blot images and **(D)** quantification of p-CHK protein levels in mice livers (*n* = 4). **(E)** Relative mRNA levels (*n* = 4) of *Wrnip1*, *Fan1*, *Nabp2*, *Ino80* and *Prpf19* in mice livers. **(F)** Representative γH2AX staining of mouse liver at 6 h post-irradiation (*n* = 3). Nuclei were stained with DAPI (blue); scale bar, 20 μm. **(G)** Quantification of persistent γH2AX foci per nucleus at 6 h post-irradiation (*n* = 3). All error bars represent SD. *p < 0.05, **p < 0.01, ***p < 0.001.

γH2AX was used as a marker of DNA damage (37). Under normal physiological conditions, almost no γH2AX foci was detected in the liver of mice (Supplementary Figure S1). To evaluate the impact of multigenerational DRF on the DNA damage response in mouse liver, mice were exposed to X-ray irradiation with a total-body dose of 1 Gy. This exposure induced pronounced γH2AX foci formation in the mouse liver, particularly in F10 mice, where a greater abundance of γH2AX signals were detected (Figures 2A,B). To further validate the hyperactivation of the upstream DNA damage response, we evaluated the phosphorylation levels of checkpoint kinase (p-CHK). Consistent with the γ H2AX accumulation, Western blot analysis revealed that irradiation-induced p-CHK expression significantly increased across successive generations, peaking in the F10 livers (Figures 2C,D). We also found the expression of DNA repair genes which lost transcriptional rhythm during multigenerational DRF was lower in the liver of F10 mice (Figure 2E). To further assess actual repair efficiency and kinetics, we tracked the resolution of γH2AX foci at 6 h post-irradiation. Consistent with the impaired expression of repair genes, F5 and F10 mice exhibited significantly higher levels of persistent γH2AX foci compared to earlier generations at this delayed time point (Figures 2F,G). Thus, multigenerational DRF not only enhances the initial susceptibility of mouse livers to genotoxic stress but also genuinely impairs their ability to clear genomic lesions over time.

### In silico validation identifies specific DNA repair genes as direct circadian clock targets

**Figure 3:**
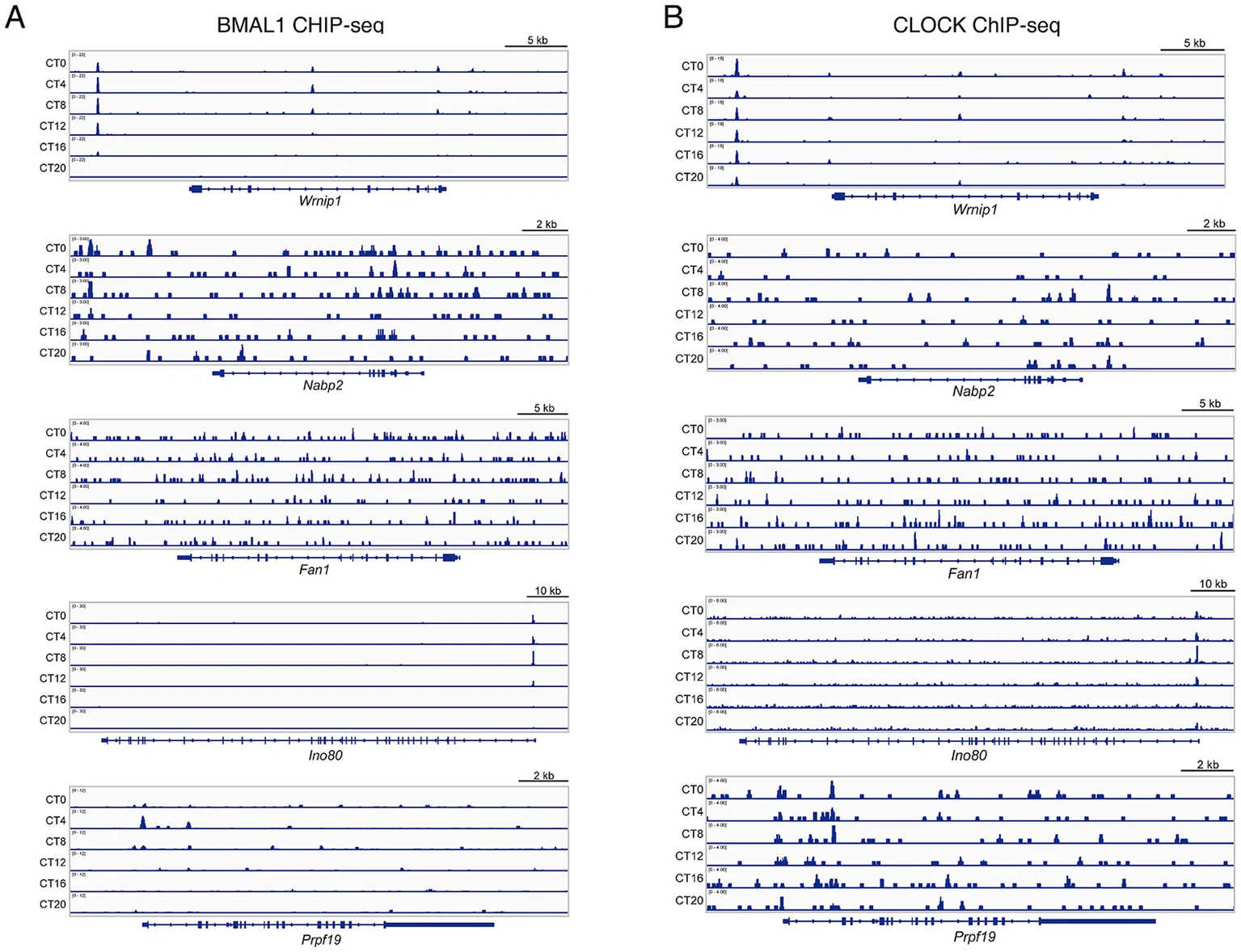
Dynamic *in vivo* recruitment of BMAL1 and CLOCK to the regulatory regions of DNA repair genes. Representative high-resolution time-course ChIP-seq tracks for **(A)** BMAL1 and **(B)** CLOCK at the promoter regions of *Wrnip1*, *Nabp2*, *Fan1*, *Ino80*, and *Prpf19* in mouse liver. Data were obtained from the publicly available GSE39860 dataset. Liver samples were collected every 4 h across a 24-h circadian cycle (from Circadian Time CT0 to CT20). The *y*-axis represents normalized read counts.

To explore the upstream regulatory mechanisms driving the loss of rhythmicity in specific DNA repair genes, an *in silico* analysis was conducted to determine whether they constitute direct targets of the core circadian clock. Initial motif prediction using the JASPAR database confirmed the presence of canonical BMAL1-CLOCK E-box motifs within the 2-kb upstream promoter regions of *Wrnip1*, *Nabp2*, *Fan1*, *Ino80*, and *Prpf19* (Supplementary Figure S2). To verify physiological binding dynamics *in vivo*, publicly available, high-resolution time-course ChIP-seq datasets from mouse liver (GSE39860) were analyzed. This temporal analysis revealed time-dependent recruitment of both BMAL1 and CLOCK to the promoters of *Ino80* and *Prpf19*, while *Wrnip1* exhibited rhythmic CLOCK binding signals (Figure 3). Further cross-validation utilizing multiple hepatic datasets from the ChIP-Atlas database corroborated the strong co-binding of BMAL1 and CLOCK at the regulatory regions of *Wrnip1*, *Ino80*, and *Prpf19* (Supplementary Figure S3). In contrast, *Nabp2* and *Fan1* lacked consistent BMAL1 co-binding signals in these specific datasets, despite possessing theoretical E-box motifs. Collectively, these functional genomic data substantiate that *Wrnip1*, *Ino80*, and *Prpf19* are direct downstream transcriptional targets of the CLOCK: BMAL1 heterodimer in the liver.

### Multigenerational DRF sensitizes liver to DNA damage induced cellular senescence

**Figure 4:**
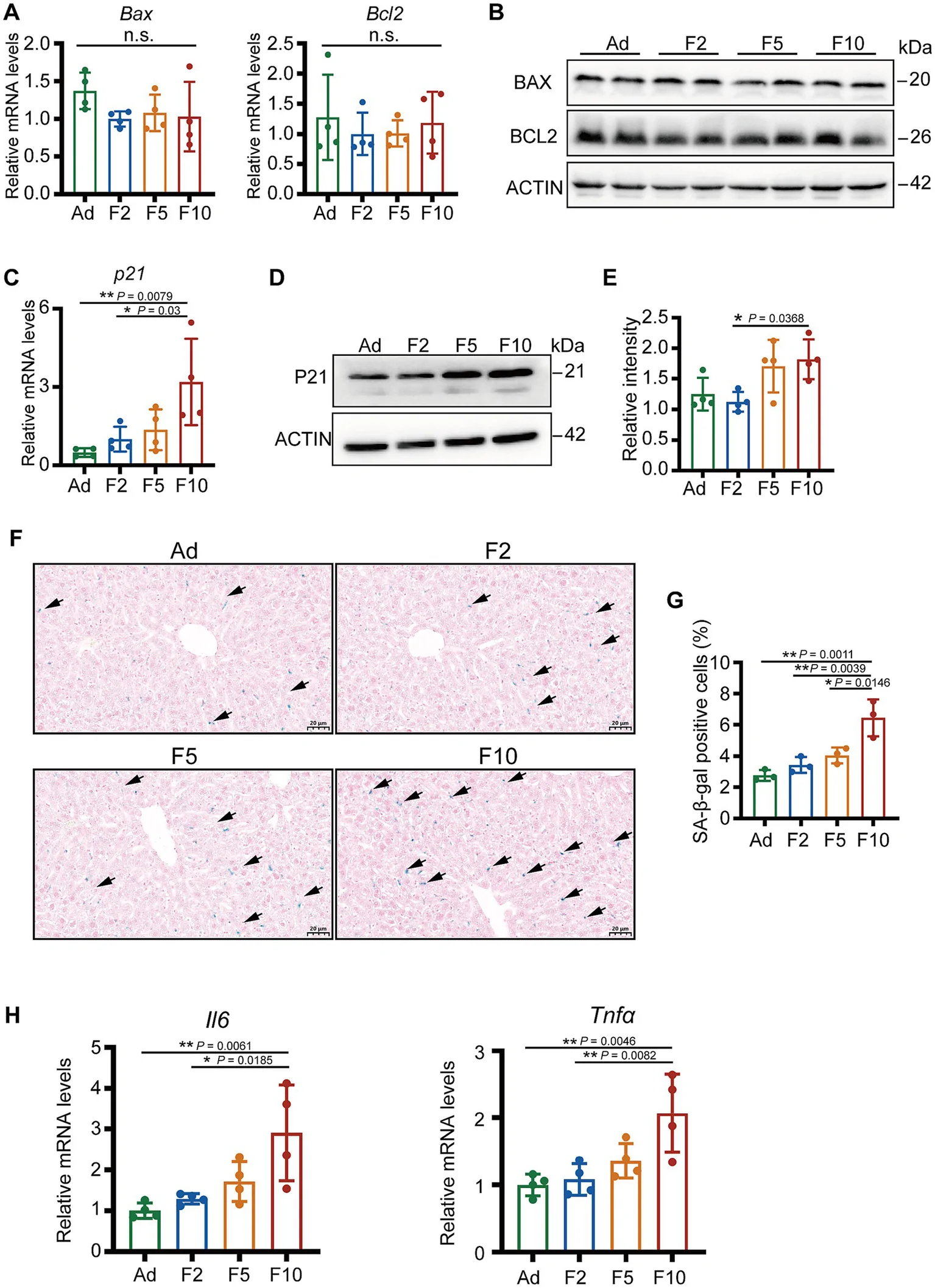
Multigenerational DRF sensitizes liver to DNA damage induced cell cycle arrest. Liver tissues were sampled 30 min after X irradiation. **(A)** Relative mRNA (*n* = 4) and **(B)** protein (*n* = 4) levels of *Bax* and *Bcl2* in mice livers. **(C)** Relative mRNA (*n* = 4) and **(D)** protein (*n* = 4) levels of *p21* in mice livers. **(E)** Quantification of p21/ACTIN protein levels (*n* = 4). **(F)** Representative SA-β-Gal staining of mouse liver (*n* = 3). Nuclei were stained with Fast Red; scale bar, 20 μm. β-Gal positive cells are localized to the arrowhead-marked region. **(G)** Quantification of SA-β-Gal positive cells (*n* = 3). **(H)** Relative mRNA (*n* = 4) levels of SASP markers *Il6* and *Tnfa*. All error bars represent SD. *p < 0.05, **p < 0.01, *n.s.*, not significant.

DNA damage can induce either cell cycle arrest or apoptosis (38). To investigate the effect of multigenerational DRF on the fate of DNA-damaged hepatic cells, the expression of cell cycle arrest and apoptosis markers was detected. Our results showed that both the mRNA and protein levels of the pro-apoptotic protein BAX and the anti-apoptotic protein BCL2 were similar among DRF groups (Figures 4A,B). However, the expression levels of cell cycle arrest marker p21 in F10 were higher than other groups (Figures 4C–E). To evaluate the delayed cellular consequences, cellular senescence was assessed by SA-β-Gal staining. We revealed a significant increase in the number of senescent cells in the livers of F10 mice (Figures 4F,G). Under non-irradiated conditions, the basic levels of β-gal positive cells were low and showed no significant differences between groups (Supplementary Figure S4). To further characterize the senescence-associated secretory phenotype (SASP) that shapes the pro-senescent hepatic microenvironment, we measured hepatic mRNA expression of canonical SASP mediators *Il6* and *Tnfa* following irradiation. Under genotoxic stress, both *Il6* and *Tnfa* transcripts were progressively upregulated across successive DRF generations, with F10 mice displaying significantly higher expression than F2 (Figure 4H). These results indicate that in response to irradiation-induced DNA damage, multigenerational DRF exacerbates stress-induced hepatocellular senescence.

## Discussions

This study reveals the significant intergenerational impacts of multigenerational DRF on the hepatic DDR. Using a multigenerational DRF mouse model, we observed that key DNA repair genes (e.g., *Wrnip1*, *Nabp2*, *Fan1*, *Ino80*, and *Prpf19*) lost their rhythmic expression and exhibited downregulated mRNA levels from the F2 to F10 generations. These genes are integral to various aspects of DNA repair, such as double-strand break repair, base excision repair, and nucleotide excision repair (21, 33–36). Consequently, F10 mice displayed an exacerbated accumulation of γH2AX foci following 1 Gy X-ray exposure, indicating a heightened susceptibility to genotoxic stress and a long-term impairment in hepatic DNA repair capacity.

Interestingly, multigenerational DRF did not alter the basal apoptosis pathway, as the expression of pro-apoptotic BAX and anti-apoptotic BCL2 remained unchanged. This is likely because the 1 Gy irradiation dose is relatively low and primarily induces cellular senescence rather than apoptosis (39). Instead, F10 livers showed significantly increased expression of the cell cycle arrest marker p21 and enhanced SA-β-gal activity following irradiation. This indicates a preferential shift towards cell cycle arrest to prevent the propagation of DNA damage (40), which ultimately promotes a senescence-prone hepatic microenvironment. Beyond cell-autonomous growth arrest, we further detected markedly increased expression of the SASP pro-inflammatory cytokines Il6 and Tnfa in irradiated F10 livers. SASP factors secreted by damaged hepatocytes act in a paracrine manner to propagate senescence signals to neighboring cells, jointly forming a senescence-prone hepatic microenvironment characterized by persistent DNA damage, permanent cell cycle arrest, and pro-inflammatory secretory activity. Collectively, our multi-marker evidence (γH2AX for DNA lesions, p21 for cell cycle arrest, SA-β-gal for senescent cell identity, and *Il6*/*Tnfa* for SASP inflammation) robustly supports the conclusion that lifelong multigenerational DRF predisposes the liver to genotoxic stress-triggered cellular senescence. This outcome provides a compelling contrast to other “two-hit” models of hepatic vulnerability. For instance, prenatal caffeine exposure (PCE) induces hepatic damage via a p53-independent pathway driven by metabolic overload and acute endoplasmic reticulum stress, resulting in direct caspase-3-mediated apoptosis without genomic instability (41). Conversely, our multigenerational DRF model compromises chronobiological defenses against external double-strand DNA damage. Rather than driving localized apoptosis, this behavioral-chronobiological stressor forces hepatocytes into a p53/p21-dependent premature senescence state. This comparison provides a valuable framework for understanding how distinct stressors—chemical-metabolic versus behavioral-chronobiological—dictate fundamentally different downstream stress-execution pathways and long-term organ health outcomes.

Dietary timing is a potent modulator of hepatic circadian rhythms. Building on our previous findings that multigenerational DRF blunts the endoplasmic reticulum (ER) stress response (11), the current study establishes a novel phenotype concerning genomic integrity. We demonstrate a mechanistic link where persistent mistimed feeding disrupts the circadian regulation of DDR genes, functionally impairing the liver’s response to genotoxic stress. This primary defect in DNA repair, potentially compounded by the attenuated ER stress response, may create a vicious cycle of accumulating genotoxic and proteotoxic damage that culminates in the pronounced aging phenotype observed in F10 mice.

To investigate the upstream mechanisms driving this circadian dysregulation, our *in silico* analysis demonstrated that *Wrnip1*, *Ino80*, and *Prpf19* are direct transcriptional targets of the CLOCK:BMAL1 heterodimer, characterized by temporal *in vivo* binding at their promoter regions. Given that the expression of core clock genes themselves remained largely unaltered in our multigenerational DRF model (11), we hypothesize that the observed heritable rhythm loss in these specific downstream targets is primarily driven by acquired epigenetic modifications (such as altered DNA methylation or H3K27ac deposition) (8, 9, 42, 43). These epigenetic alterations, induced by persistent mistimed feeding, may physically obscure the validated E-box elements within the promoters of these DNA repair genes, thereby uncoupling them from normal circadian regulation. Conversely, the rhythmicity of *Nabp2* and *Fan1* may be governed by secondary clock-controlled transcription factors, which warrants further experimental exploration. However, we acknowledge a major limitation of our current study: we lack direct epigenetic evidence, such as bisulfite sequencing or chromatin immunoprecipitation followed by quantitative PCR (ChIP-qPCR), to definitively substantiate this mechanistic link. Furthermore, from a broader perspective, our current experimental design cannot provide a concrete explanation as to why these phenotypic and transcriptomic defects accumulate and manifest so dramatically precisely at the F10 generation. It remains entirely unknown whether this cumulative effect is driven primarily by maternal lineages, paternal lineages, or interacting environmental factors within the breeding cohorts. Employing cross-fostering or “release” models, paired with targeted transgenerational epigenetic profiling, will be crucial for future studies to bridge the gap between our functional observations and the underlying molecular mechanisms, and to determine how these phenotypic changes are transmitted.

In conclusion, multigenerational DRF disrupts the circadian coordination of DNA repair and sensitizes the liver to genotoxic stress, which can accelerate liver aging. Because aging is a critical risk factor for hepatic diseases, these findings emphasize that considering “when we eat” is as critical as “what we eat” in preventing age-related liver pathologies.

## Data Availability

The data analyzed in this study is available in the NCBI BioProject repository (https://www.ncbi.nlm.nih.gov/bioproject) under accession number PRJNA932189.

## Supplementary material

SUPPLEMENTARY FIGURE S1 Representative γH2AX staining (red) of mouse liver (*n* = 3). Nuclei were stained with DAPI (blue); scale bar, 20 μm.

SUPPLEMENTARY FIGURE S2 *In silico* prediction of BMAL1-CLOCK binding motifs within the promoter regions of DNA repair genes. **(A)** Sequence logo representing the canonical BMAL1 binding motif obtained from the JASPAR database. **(B)** Summary tables of the predicted BMAL1 (*Arntl*) binding sites within the 2-kb transcription start site (TSS)-upstream promoter regions of *Wrnip1*, *Nabp2*, *Fan1*, *Ino80*, and *Prpf19*. The predictions were generated using the JASPAR database, detailing the start/end positions, strand orientations, relative scores, and predicted sequences. **(C)** Schematic representation of the 2-kb upstream promoter regions. The specific nucleotide sequences containing the highest-scoring predicted E-box motifs for each respective gene are highlighted in red.

SUPPLEMENTARY FIGURE S3 Cross-validation of BMAL1 and CLOCK binding at the regulatory regions of DNA repair genes using the ChIP-Atlas database. Representative genome browser tracks obtained from the ChIP-Atlas public database, illustrating the integrated binding signals of BMAL1 (annotated as *Arntl*) and CLOCK across multiple mouse liver ChIP-seq datasets. The canonical core clock gene *Per1* is presented at the top as a positive control, demonstrating robust CLOCK: BMAL1 co-binding. The color of the peak blocks indicates the statistical significance value [−10*Log10(MACS2 Q-value)] calculated by the MACS2 peak-caller. A significance threshold was set at 50 (*Q*-value < 1E-05), and the significance levels are color-coded as follows: blue (50), cyan (250), green (500), yellow (750), and red (>1,000).

SUPPLEMENTARY FIGURE S4 Representative SA-β-Gal staining of mouse liver (*n* = 3). Nuclei were stained with Fast Red; scale bar, 20 μm. β-Gal positive cells are localized to the arrowhead-marked region.
